# Pulmonary valve perforation with multiple cardiac anomalies: a case report

**DOI:** 10.1186/s12872-022-02595-9

**Published:** 2022-04-09

**Authors:** Yan Chen, Huanhuan Gao, Yun Mou, Zhelan Zheng

**Affiliations:** Echocardiography and Vascular Ultrasound Center, The First Affiliated Hospital, College of Medicine, Zhejiang University, No.79, Qing-Chun Road, Hangzhou, 310003 China

**Keywords:** Pulmonary valve, Infective endocarditis, Echocardiography, Case report

## Abstract

**Background:**

Large pulmonary valve perforation, which is rarely seen with infective endocarditis, general atrophy, or congenital fenestration, often leads to potentially fatal outcomes, including heart failure.

**Case presentation:**

Transthoracic and transesophageal echocardiographic evaluation of a 69-year-old woman revealed a severely eccentric pulmonary regurgitation with concomitant pulmonary valve stenosis, patent ductus arteriosus, patent foramen ovale, and pulmonary artery aneurysm. In the operation, a large perforation was found in the pulmonary valve leaflet. She underwent complicated surgery that involved closure of the congenital heart defects and replacement of a pulmonary valve with successful results. But the cause of her pulmonary valve perforation remained undetermined.

**Conclusion:**

This case highlights two important points: the need for timely management of congenital heart disease and being aware of the possibility of pulmonary valve perforation, which in this case was indicated by an eccentric pulmonary regurgitant jet seen on echocardiography.

## Background

Pulmonary valve (PV) perforation can be attributed to an acquired or congenital lesion. Infective endocarditis (IE) is the common etiology for cardiac valve perforation, although PV IE is extremely rare, occurring in < 2% of patients with IE [1]. Fenestration of semilunar valves with general atrophy, which gradually progresses with age, is generally seen in older people [2]. Congenital fenestration of the PV should be considered an abnormality that often occurs concomitantly with PV stenosis.

We report a case of large perforation of the pulmonary leaflet along with multiple congenital anomalies in an older woman.

## Case presentation

A 69-year-old woman presented to our hospital with a 5-month history of chest tightness, palpitation, nausea, and fatigue during activity. The symptoms were alleviated at rest. Chest computed tomography that had been performed in a local hospital showed cardiomegaly without a pulmonic lesion. She had a 9-year history of hypertension and declared there had been no recent continuous fever, except for “flu,” for which she had received general treatment in a local clinic 6 months previously.

**Fig. 1:**
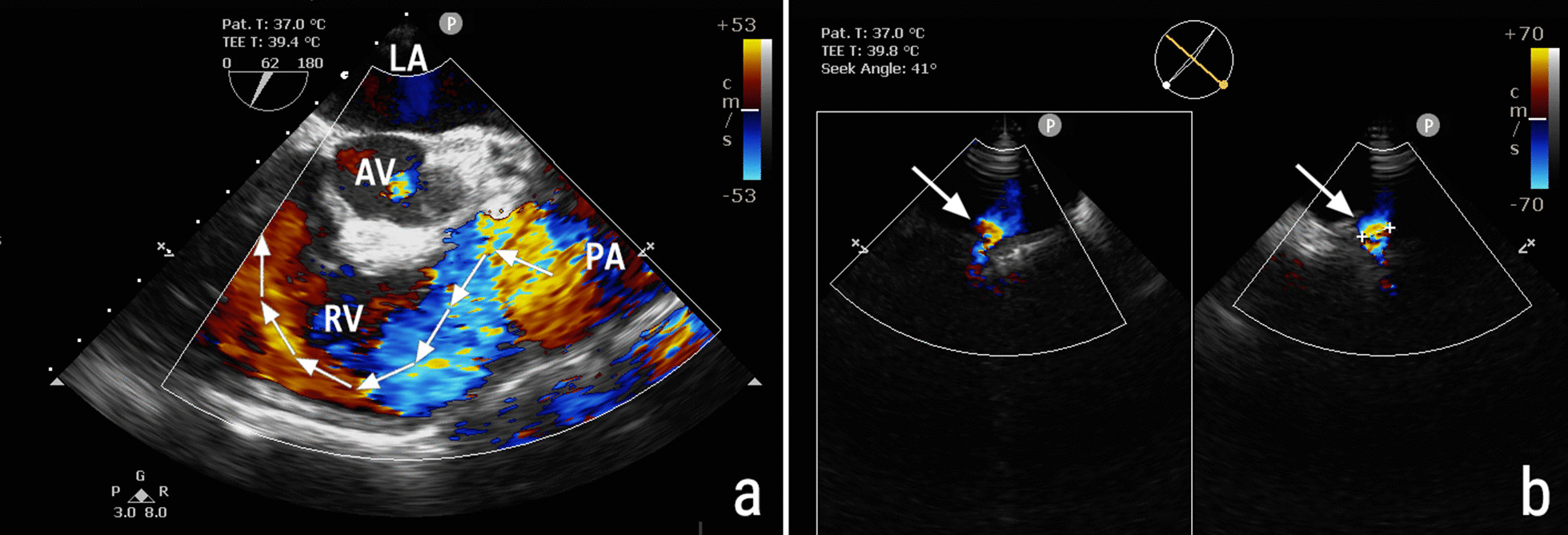
Transesophageal echocardiography. **a** Color Doppler imaging shows severe pulmonic valve regurgitation with an eccentric jet. The jet’s path is marked with seven arrows. **b** Color Doppler biplane imaging shows patent ductus arteriosus (*arrow*). *LA* left atrium, *AV* aortic valve, *RV* right ventricle, *PA* pulmonary artery

Further evaluation revealed that the patient was afebrile with stable vital signs. Transthoracic echocardiography (TTE) showed enlargement of the left ventricle (60 mm diameter during diastole) with moderate aortic regurgitation due to valve degeneration, dilatation of the right heart without right ventricular dysfunction, and moderate tricuspid regurgitation (tricuspid annulus diameter 45 mm). TTE also revealed PV stenosis with a peak velocity of 4 m/s and patent foramen ovale (PFO). Transesophageal echocardiography (TEE) was performed because of poor visualization of the pulmonary artery on TTE. TEE had revealed a large main pulmonary artery (diameter 56 mm), severe pulmonary regurgitation with vena contracta of 15 mm (Fig. 1a), patent ductus arteriosus (PDA) with a diameter of 7 mm (Fig. 1b), and a thickened, dome-shaped PV with no attached vegetation. The patient was diagnosed with multiple congenital heart diseases—PV stenosis, PDA, PFO—accompanied by degeneration of the aortic valve. The cause of her pulmonic regurgitation, however, remained undetermined.

**Fig. 2:**
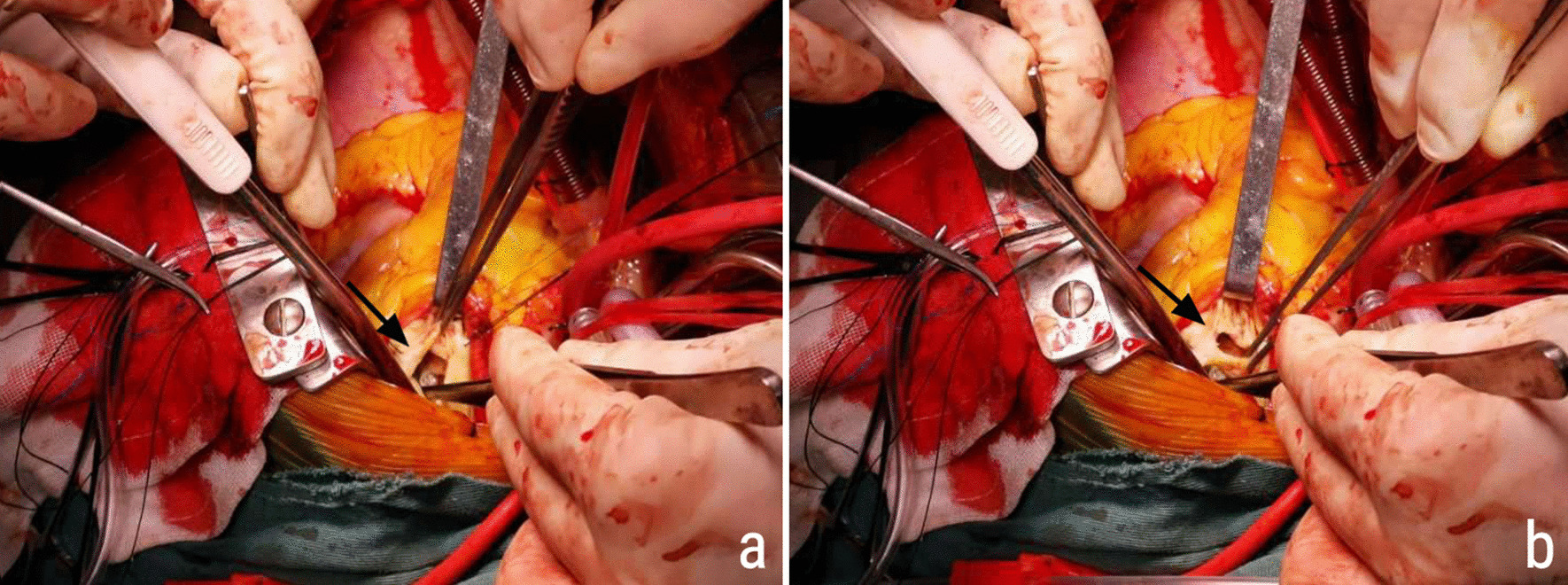
Intraoperative photographs of the pulmonary valve. **a** Pulmonary valve stenosis with restrictive orifice (*arrow*). **b** Large perforation (*arrow*) of the pulmonary valve

Cardiac surgery was scheduled after preoperative tests showed that the white blood cell count and C-reactive protein level were within normal ranges. Intraoperatively, the surgeon found an approximately 2 cm^2^ perforation in the PV leaflet (Fig. 2), and the pulmonary artery had a rough surface around the PDA orifice with no vegetation. The patient successfully underwent a complicated procedure involving closure of the PDA and PFO, replacement of the PV and aortic valve with Medtronic bioprosthetic valves (#27 and #25, respectively), and tricuspid valvuloplasty with a #30 Solo ring under cardiopulmonary bypass. She was discharged on postoperative day 10. At the 3-month follow-up, the patient remained asymptomatic, and TTE revealed good function of the bioprosthetic valves.

## Discussion and conclusions

This is an extremely rare case of a large PV perforation with multiple cardiac anomalies. The common etiologies of PV perforation include IE, general atrophy, congenital fenestration, and surgical procedures that include intentional perforation during cardiac catheterization studies. Our patient had no special medical history, and the exact cause of the perforation remained unclear.

IE could lead to PV perforation, although IE occurs far less often in the PV than in other native valves, accounting for only 1.5–2.0% of all IE cases [1]. The principal predisposing factors for PV IE include intravenous drug abuse, alcoholism, and sepsis due to catheter-related infections [3]—none of which was applicable in our case. According to the Duke criteria, a positive blood culture and history of fever are important factors for diagnosing IE. Preoperatively, however, our patient had no fever, and inflammatory markers were normal. Hence, no blood cultures were performed. We considered the possibility of IE mainly based on the surgical findings of PV damage and the rough surface of the pulmonary artery around the PDA orifice. An unrepaired PDA with turbulent shunt flow can cause endothelial injury and subsequent seeding of pathogens on the injured endothelium [4]. Furthermore, PV stenosis may allow longer duration of blood contact with the endocardium, which may lead to pathological changes in bacterial deployment and proliferation, resulting in IE [5]. In the present case, she had two predisposing factors for IE development: PDA and PV malformation. In addition, the PDA jet was directed toward the affected leaflet, which could contribute to valve injury. As this high flow may cause endothelial disruption in loci with turbulence during bacteremia. The PV IE then probably caused tissue necrosis of the leaflet, which resulted in ulceration, leading to perforation of the valve and eventual regurgitation.

Congenital fenestration should be considered as the patient had concomitant cardiac anomalies, especially PV stenosis. Fenestration on the leaflet can alleviate the hemodynamic effect of stenosis. Although it increases the volume of the right ventricle with PV regurgitation, patients often have clinical tolerance due to lower pressure in the right heart. PV fenestration with general atrophy could not be completely excluded as it was reported to be present in 55% of 342 patients 60–70 years of age at autopsy after excluding the usual congenital heart diseases. Additionally, there seems to be an increase in the average size of the opening with advancing age [2].

Echocardiography is the first-line imaging method for the diagnosis of PV lesion and perforation. However, TTE is diagnostic only in the cases with clear sonogram. TEE is challenging because of the limited visualization resulting from the large distance between the PV and the echo probe. Furthermore, it is more difficult to document a perforation in the PV directly, compared with diagnosis in the aortic and mitral valves, where it is diagnosed using three-dimensional echocardiography. We believe that the presence of a moderate-to-severe, eccentric, pulmonary regurgitant jet probably suggests valve perforation. Meanwhile, multimodal imaging improves the identification of PV lesion in patients with negative TTE or TEE findings [6].

Regardless of the etiology of PV perforation in our case, PV replacement was inevitable due to the leaflet damage incurred. Even for patients with uncontrollable PV IE, PV replacement has been proved to be an effective treatment, with excellent early outcomes and good late results [5]. The enlarged, dilated left ventricle in our patient was thought to be caused by the combined impact of the PDA and moderate aortic regurgitation, which caused left ventricular volume overload. PDA closure and aortic valve replacement were performed to address these issues.

Although the most probable cause of pulmonary artery aneurysm is post-stenotic dilation, an aneurysm can arise in the pulmonary artery secondary to various etiologies (e.g., congenital heart disease, infection, vasculitis) or interplay with multiple factors. Its management strategies remain variable, depending on the relevant conditions, such as the underlying disease and its etiology [7]. Although surgery is the main therapy for pulmonary artery aneurysms, evidence suggesting an absolute diameter threshold for surgery of the main pulmonary artery is still lacking. Kreibich et al. [8] suggested surgery in adults with pulmonary trunk aneurysms measuring > 5.5 cm in accordance with the guidelines for aortic disease. In our case, the surgeons chose not to replace the aneurysmal site for several reasons. One was that the procedure undertaken included PDA closure and PV replacement, which had addressed the cause of the pulmonary artery dilation. The second reason was that the patient had no history of related diseases, such as pulmonary disease, connective tissue abnormalities, autoimmune disease, or vasculitis, that could contribute to aneurysm progression or rupture. The third reason was that the pulmonary artery is under only low pressure, unlike the ascending aorta. Additionally, they considered the patient’s age and the advantage of keeping her own blood vessel. Hence, the main pulmonary artery was not replaced.

The present case shows the rare appearance of concomitant PDA, PFO, PV stenosis with perforation, and aneurysm in the pulmonary artery. Although the etiology of the PV perforation remains undetermined, this unusual case emphasizes two critical points (1) the importance of timely treatment of congenital heart disease and (2) apparent PV regurgitation with an eccentric jet probably suggests the presence of leaflet perforation. As echocardiographers, we should be aware of PV IE as a cause of eccentric pulmonary regurgitation, and a complete echocardiographic evaluation of right heart is important, especially when the patient has had a recent episode of fever. A combination of TTE and TEE is necessary to evaluate probable pulmonary IE and concomitant multiple cardiac conditions.

## Data Availability

The datasets used in the case are available from the corresponding author upon reasonable request.

## Abbreviations

PV: Pulmonary valve
IE: Infective endocarditis
TTE: Transthoracic echocardiography
PFO: Patent foramen ovale
TEE: Transesophageal echocardiography
PDA: Patent ductus arteriosus
LA: Left atrium
AV: Aortic valve
RV: Right ventricle
PA: Pulmonary artery
